# A Case of Intraductal Tubulopapillary Neoplasm of the Pancreas Requiring Superior Mesenteric Vein–Portal Vein Axis Resection after Systemic Chemotherapy

**DOI:** 10.70352/scrj.cr.26-0181

**Published:** 2026-09-10

**Authors:** Haruto Sone, Yasuhisa Ando, Jun Kozai, Dongping Feng, Hiroshi Taketani, Hiroyuki Matsukawa, Bunpei Nishiura, Akihiro Kondo, Kiyoyuki Kobayashi, Hironobu Suto, Takayoshi Kishino, Ryou Ishikawa, Yoichi Yamashita, Minoru Oshima, Hideki Kobara, Keiichi Okano

**Affiliations:** 1 Postgraduate Clinical Training Center, Kagawa University, Miki-cho, Kagawa, Japan; 2 Department of Gastroenterological Surgery, Faculty of Medicine, Kagawa University, Miki-cho, Kagawa, Japan; 3 Department of Gastroenterology and Neurology, Faculty of Medicine, Kagawa University, Miki-cho, Kagawa, Japan; 4 Department of Diagnostic Pathology, Faculty of Medicine, Kagawa University, Miki-cho, Kagawa, Japan; 5 Department of Cardiovascular Surgery, Faculty of Medicine, Kagawa University, Miki-cho, Kagawa, Japan

**Keywords:** intraductal tubulopapillary neoplasm, intraductal tubulopapillary neoplasm (ITPN), chemotherapy, pancreas, superior mesenteric vein–portal vein axis resection

## Abstract

**INTRODUCTION:**

Intraductal tubulopapillary neoplasm (ITPN) is a rare intraductal pancreatic tumor, accounting for approximately 3% of all intraductal neoplasms. ITPN is characteristically paucimucinous and lacks low-grade (adenomatous) components. Since reported cases of ITPN remain limited, its prognosis, prognostic factors, and role of perioperative chemotherapy are not well defined.

**CASE PRESENTATION:**

A 64-year-old man presented with loss of appetite and abdominal pain in year X–1. Endoscopic US–guided fine-needle aspiration (EUS-FNA) was performed, and the findings showed atypical epithelial cells suspicious for adenocarcinoma. The lesion was radiologically assessed as borderline-resectable pancreatic head cancer with portal venous involvement. Curative-intent resection was attempted at the referring hospital; however, the tumor was judged intraoperatively to be locally unresectable because safe vascular dissection around the superior mesenteric vein (SMV) was difficult. Choledochojejunostomy, cholecystectomy, and gastrojejunostomy were performed for obstructive jaundice and duodenal stenosis. From October X–1, the patient received gemcitabine plus nab-paclitaxel, followed by tegafur/gimeracil/oteracil. The maximum tumor diameter decreased from 60 to 50 mm, and the imaging response to systemic chemotherapy was classified as stable disease according to the Response Evaluation Criteria in Solid Tumors (RECIST) version 1.1, without distant progression. In May X, the patient was referred to our department for consideration of surgical treatment. Repeat EUS-FNA strongly suggested ITPN. Subtotal stomach-preserving pancreatoduodenectomy with SMV–portal vein (PV) axis resection and reconstruction was performed using a right common femoral vein graft. Postoperative right deep femoral vein thrombosis was managed with anticoagulation. The patient was discharged on POD 16, and follow-up CT 1 year after surgery confirmed thrombus resolution. Histopathological examination confirmed invasive ITPN without lymph node metastasis or vascular invasion. The histological treatment effect was classified as grade 1a according to the Japanese classification of pancreatic carcinoma by the Japan Pancreas Society, eighth edition, and as grade I according to the Evans classification. Adjuvant chemotherapy was not administered. The patient remained disease-free 29 months after surgery.

**CONCLUSIONS:**

This case highlights the importance of multidisciplinary re-evaluation in pancreatic tumors initially managed as pancreatic ductal adenocarcinoma, particularly when an unusual histology such as ITPN is suspected.

## Abbreviations

ACC
: acinar cell carcinoma
CA19-9
: carbohydrate antigen 19-9
CEA
: carcinoembryonic antigen
CFV
: common femoral vein
DUPAN-2
: Duke pancreatic antigen-2
DWI
: diffusion-weighted imaging
EUS-FNA
: endoscopic US–guided fine-needle aspiration
FDG
: ^18^F-fluorodeoxyglucose
IHC
: immunohistochemistry
IPC
: intermittent pneumatic compression
ITPN
: intraductal tubulopapillary neoplasm
MRCP
: magnetic resonance cholangiopancreatography
MUC1/2/5AC/6
: mucin core proteins 1/2/5AC/6
PV
: portal vein
RECIST
: Response Evaluation Criteria in Solid Tumors
S-1
: tegafur/gimeracil/oteracil
SFJ
: saphenofemoral junction
SMA
: superior mesenteric artery
SMV
: superior mesenteric vein
SPAN-1
: sialylated pancreatic antigen-1
SUVmax
: maximum standardized uptake value
TNM
: tumor–node–metastasis
UICC
: Union for International Cancer Control

## INTRODUCTION

ITPN is a rare pancreatic intraductal tumor entity originally described in Japan.^1)^ Owing to the small number of reports, key issues, including the prognosis and role of perioperative chemotherapy, remain uncertain. Herein, we report a patient with pancreatic head ITPN who underwent curative-intent resection after systemic chemotherapy.

## CASE PRESENTATION

A 64-year-old man presented to a referral hospital with loss of appetite and abdominal pain. His medical history included benign prostatic hyperplasia. Initial blood tests revealed elevated levels of cholestatic enzymes with predominantly direct hyperbilirubinemia (aspartate aminotransferase, 226 U/L; alanine aminotransferase, 288 U/L; alkaline phosphatase, 640 U/L; direct bilirubin, 0.9 mg/dL; total bilirubin, 1.1 mg/dL). CT findings revealed a pancreatic head mass compressing the duodenum. EUS-FNA of the pancreatic head lesion revealed atypical epithelial cells suspicious for adenocarcinoma; the differential diagnosis included invasive ductal carcinoma and ITPN. Considering the clinical presentation, invasive ductal carcinoma was favored.

**Fig. 1:**
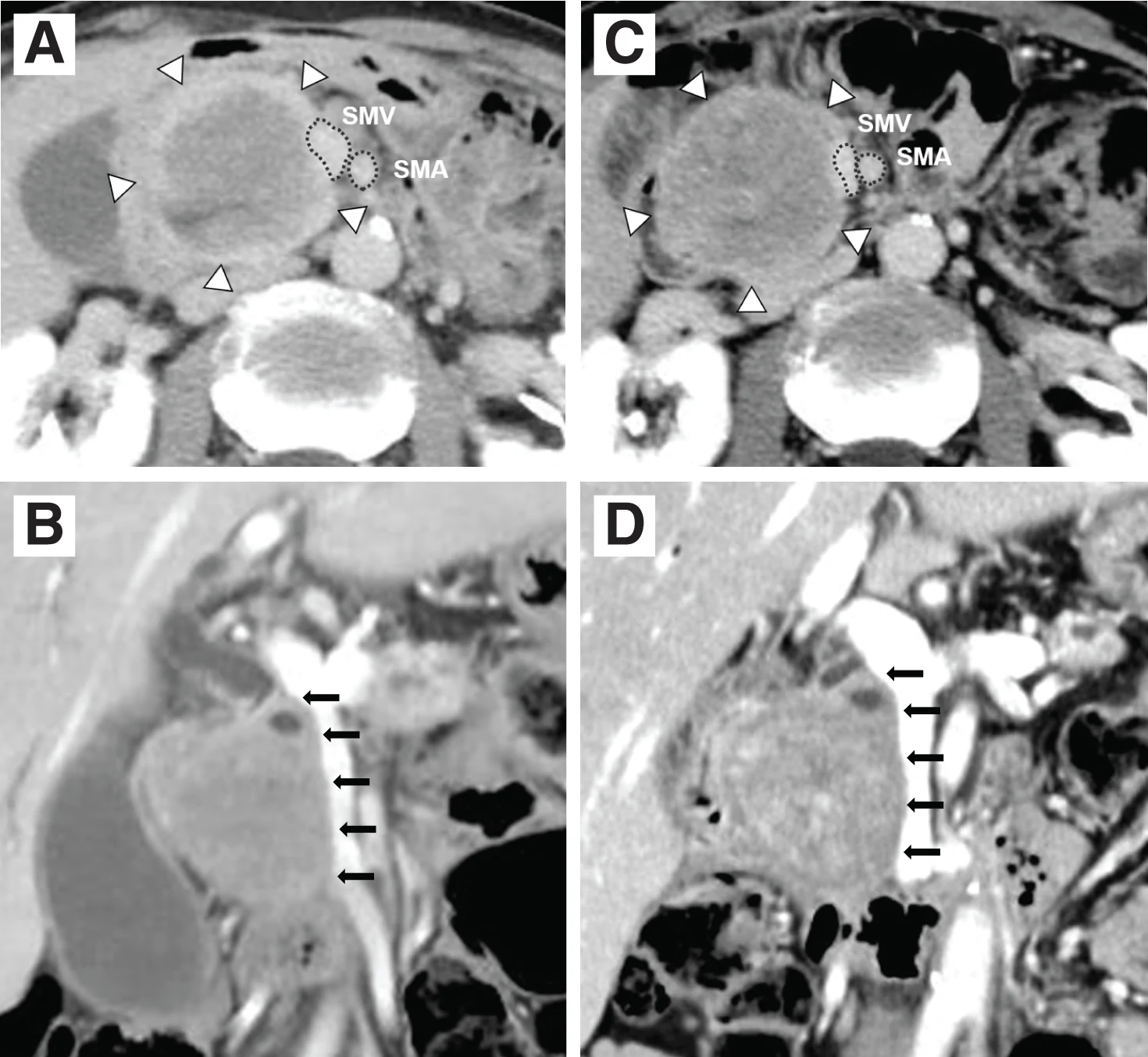
Contrast-enhanced CT before and after systemic chemotherapy. (**A**) Axial contrast-enhanced CT before systemic chemotherapy shows a 60-mm mass in the pancreatic head abutting the SMV–PV axis. (**B**) Coronal contrast-enhanced CT before systemic chemotherapy shows long-segment contact between the tumor and the SMV–PV axis, measuring approximately 50 mm in the craniocaudal direction. (**C**) Axial contrast-enhanced CT after systemic chemotherapy shows a modest decrease in tumor size to 50 mm, with persistent long-segment contact with the SMV–PV axis. (**D**) Coronal contrast-enhanced CT after systemic chemotherapy shows residual long-segment contact with the SMV–PV axis. The contact length on coronal images remained approximately 50 mm, showing no substantial interval change. The imaging response to systemic chemotherapy was classified as stable disease according to RECIST version 1.1. SMA, superior mesenteric artery; SMV, superior mesenteric vein; PV, portal vein; RECIST, Response Evaluation Criteria in Solid Tumors

At the referring hospital, the tumor was radiologically assessed as borderline resectable pancreatic cancer with portal venous involvement. Pre-treatment contrast-enhanced CT showed long-segment solid tumor contact with the SMV–PV axis, measuring approximately 50 mm in the craniocaudal direction on coronal images, with more than 180° circumferential tumor–vessel contact and compression-related deformity of the SMV (**Fig. 1A** and **1B**). These findings were considered consistent with borderline-resectable pancreatic cancer with portal venous involvement. The tumor showed only minimal contact with the SMA, without arterial encasement, and no contact with the celiac axis or common hepatic artery.

Based on this radiological assessment, curative-intent resection was attempted. During the initial operation, no peritoneal dissemination or liver metastasis was identified. However, the tumor was judged unresectable because dense adhesion to the middle colic artery and vein and marked tumor-associated induration around the retropancreatic portion of the SMV near the inferior border of the pancreas made safe vascular dissection difficult. Therefore, the planned curative resection was not performed, and choledochojejunostomy, cholecystectomy, and gastrojejunostomy were performed for biliary obstruction and duodenal stenosis.

Details of systemic chemotherapy are summarized in **Table 1**. Gemcitabine plus nab-paclitaxel was administered as gemcitabine 1200 mg/body and nab-paclitaxel 150 mg/body, each corresponding to 80.5% of the planned dose per administration. The day 8 administration was delayed to day 10 because of grade 3 neutropenia, and this regimen was discontinued after the first course because of treatment-related adverse events and difficulty tolerating continued inpatient chemotherapy. S-1 was subsequently administered at 80 mg/day on a 2-weeks-on/1-week-off schedule for 9 courses without dose reduction, treatment interruption, or adverse events. Imaging assessments during S-1 therapy showed no apparent disease progression at the referring hospital, and the final imaging response to systemic chemotherapy was classified as stable disease according to RECIST version 1.1. In May X, the patient was referred to our department for consideration of surgical treatment.

**Table 1:** Systemic chemotherapy administered before curative-intent resection

Variable	Gemcitabine plus nab-paclitaxel	S-1
Actual dose and schedule	Gemcitabine 1200 mg/body and nab-paclitaxel 150 mg/body on days 1 and 10 of a 3-week course; each dose corresponded to 80.5% of the planned dose per administration	80 mg/day orally, 2 weeks on and 1 week off
Treatment duration	1 course	9 courses
Dose modification/interruption	Day 8 administration was delayed to day 10 because of grade 3 neutropenia; treatment was discontinued after 1 course	No dose reduction or treatment interruption
Adverse events	Grade 1 fever, grade 2 constipation, and grade 3 neutropenia	None
Response assessment	Not assessed independently after 1 course	No apparent progression during S-1 therapy; final response to systemic chemotherapy was stable disease according to RECIST version 1.1

Percentages for gemcitabine plus nab-paclitaxel indicate the administered dose relative to the planned dose per administration, not a calculated cumulative relative dose intensity. S-1 was administered as planned without dose reduction or treatment interruption.

RECIST, Response Evaluation Criteria in Solid Tumors; S-1, tegafur/gimeracil/oteracil

CEA and CA19-9 were not elevated before or after systemic chemotherapy (pre-treatment: CEA, 4.06 ng/mL; CA19-9, <2.00 U/mL; post-treatment: CEA, 2.80 ng/mL; CA19-9, <2.00 U/mL). DUPAN-2 was not measured before the initiation of systemic chemotherapy at the referring hospital. At presentation to our institution after systemic chemotherapy, SPAN-1 was <10 U/mL, DUPAN-2 was 114 U/mL, and elastase-1 was <40 ng/mL.

**Fig. 2:**
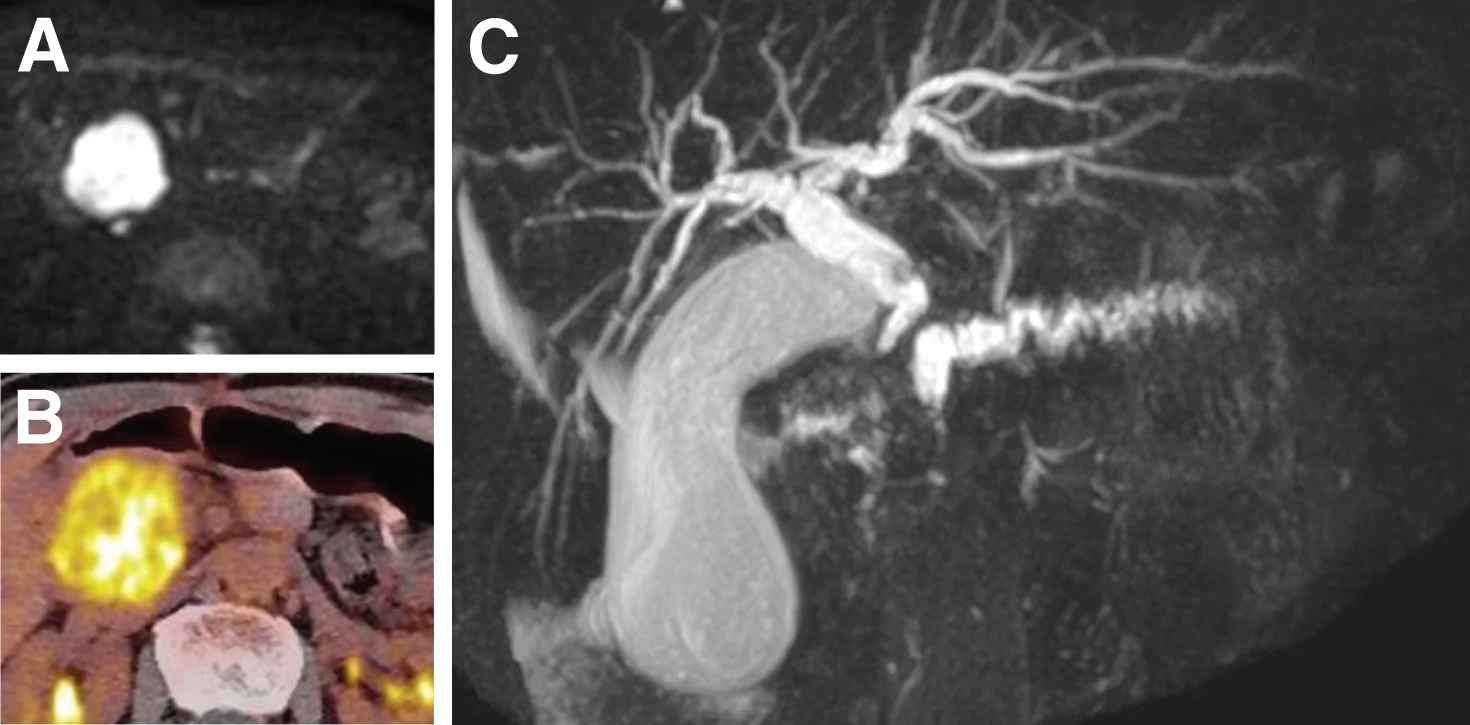
Multimodal imaging findings. (**A**) Diffusion-weighted MRI showing high signal intensity in the pancreatic head mass. (**B**) FDG-PET after systemic chemotherapy revealing abnormal uptake in the pancreatic head lesion (SUVmax 6.58). (**C**) MRCP shows a dilated main pancreatic duct with an abrupt interruption. FDG, ^18^F-fluorodeoxyglucose; SUVmax, maximum standardized uptake value; MRCP, magnetic resonance cholangiopancreatography

After systemic chemotherapy, the maximum tumor diameter decreased from 60 to 50 mm, corresponding to a 16.7% reduction (**Fig. 1A** and **1C**). No new distant metastasis was detected. The length of tumor–vessel contact with the SMV–PV axis on coronal CT was approximately 50 mm both before and after systemic chemotherapy, showing no substantial interval change (**Fig. 1B** and **1D**). According to RECIST version 1.1, the imaging response to systemic chemotherapy was classified as stable disease. The lesion continued to abut the SMV–PV axis with broad venous contact and compression-related deformity of the SMV, raising concern for venous involvement. MRI revealed high signal intensity within the mass on DWI (**Fig. 2A**). The SUVmax was 6.1 before systemic therapy and 6.58 on preoperative imaging (**Fig. 2B**); no nodal or distant uptake was identified. MRCP findings showed a dilated main pancreatic duct with an abrupt interruption, consistent with complete obstruction by an intraductal tumor (**Fig. 2C**).

EUS-FNA rebiopsy at our institution demonstrated tumor cells with round to ovoid nuclei proliferating in papillary and tubular architectures. Periodic acid–Schiff staining was negative, supporting the absence of mucin production. ITPN was suspected based on IHC. Based on the preoperative workup, we diagnosed the patient with pancreatic ITPN (ycT3N0M0, Stage IIA according to the UICC TNM 8th edition).^2)^

**Fig. 3:**
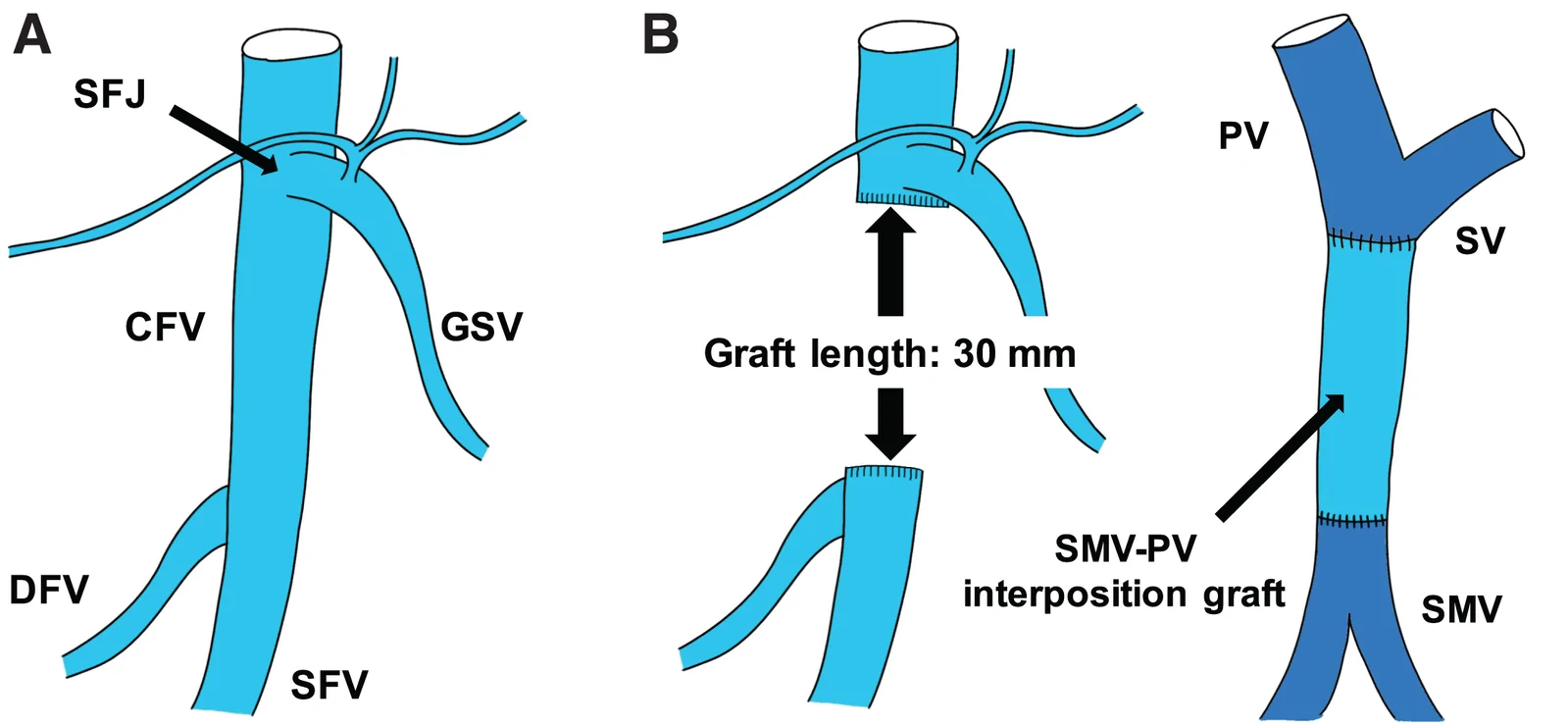
Schematic illustration of SMV–PV axis reconstruction using a CFV graft. (**A**) A 30-mm segment of the right CFV was harvested after identification and preservation of the SFJ and the confluence of the superficial femoral and deep femoral veins. The proximal and distal donor venous stumps were each closed with double-layer over-and-over sutures using 5-0 polypropylene (Prolene). (**B**) The 30-mm CFV graft was used as an SMV–PV interposition graft. CFV, common femoral vein; DFV, deep femoral vein; GSV, great saphenous vein; PV, portal vein; SFJ, saphenofemoral junction; SFV, superficial femoral vein; SMV, superior mesenteric vein; SV, splenic vein

The patient underwent subtotal stomach-preserving pancreatoduodenectomy. During laparotomy, the anterior surface of the pancreatic head revealed an exposed tumor covered by a capsule. No liver metastases were identified on intraoperative inspection. Intraoperative frozen-section analysis of peritoneal lavage cytology and para-aortic lymph node sampling was negative for malignancy. Thus, curative resection was considered feasible. As the tumor was densely adherent to the SMV–PV axis over approximately 5 cm and safe separation was considered difficult, SMV–PV axis resection and reconstruction were planned. Since the diameter of the right great saphenous vein was inadequate for venous reconstruction, the right CFV was selected as an autologous graft. After identification and preservation of the SFJ and the confluence of the superficial femoral and deep femoral veins, a 3-cm segment of the right CFV was harvested. The proximal and distal donor venous stumps were each closed with double-layer over-and-over sutures using 5-0 polypropylene (**Fig. 3A**). The CFV graft was used for SMV–PV axis reconstruction after venous resection (**Fig. 3B**). To prevent postoperative donor-limb complications, IPC with a foot pump was started on POD 0, and heparin was administered at 5000 units/day from POD 1. Anticoagulation was switched to edoxaban 15 mg/day from POD 5. Postoperative contrast-enhanced CT showed thrombosis of the right deep femoral vein; therefore, edoxaban was continued for 1 year. Follow-up CT 1 year after surgery confirmed resolution of the thrombus, and anticoagulation was discontinued.

**Fig. 4:**
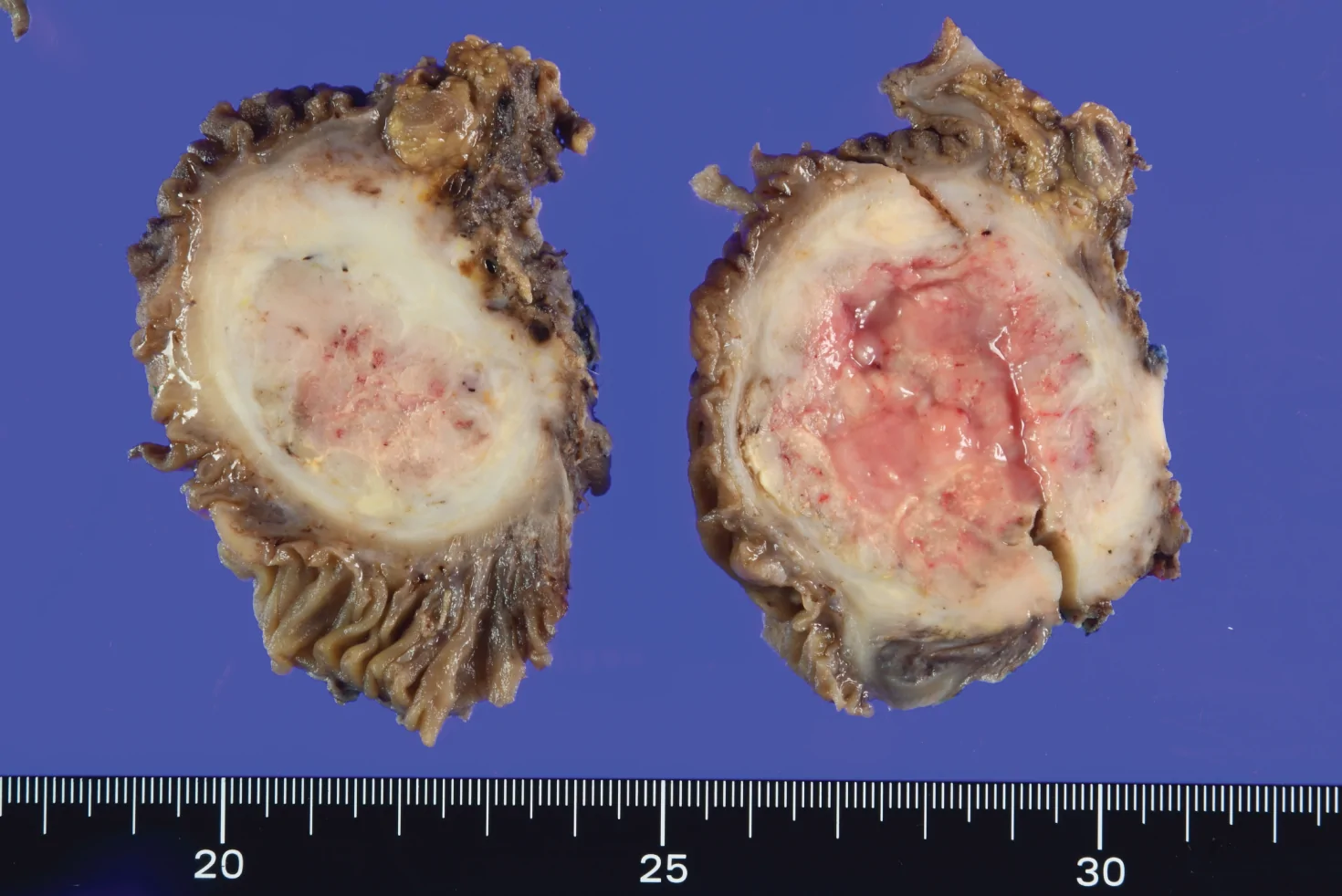
Gross appearance of the resected specimen. The resected specimen showed a solid, nodular tumor with central necrosis in the pancreatic head.

**Fig. 5:**
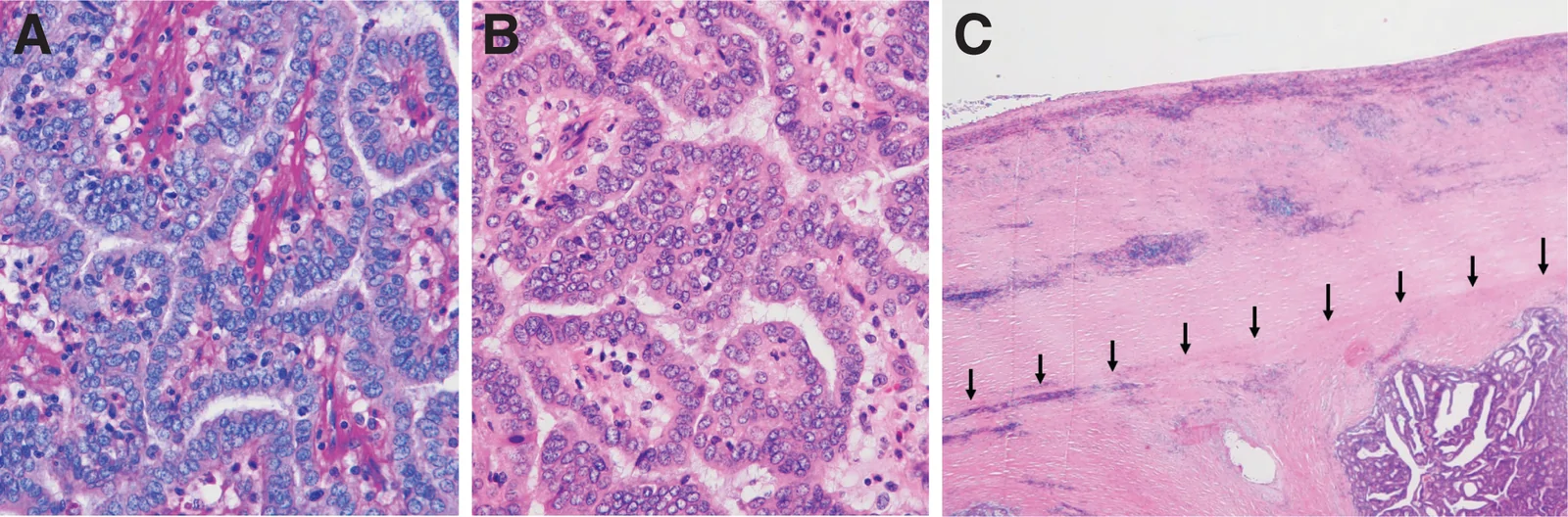
Histopathology of ITPN and SMV adhesion. (**A**) Hematoxylin and eosin staining showing tumor cells proliferating in tubular and papillary architectures with uniformly high-grade atypia. (**B**) PAS staining demonstrating absence of mucin (PAS-negative), supporting the diagnosis of ITPN. (**C**) Fibrous adhesion between the tumor and the concomitantly resected SMV, without true venous wall invasion. ITPN, intraductal tubulopapillary neoplasm; PAS, periodic acid–Schiff; SMV, superior mesenteric vein

Gross pathological findings revealed a solid nodular tumor in the pancreatic head with central necrosis (**Fig. 4**). Histologically, the tumor cells formed papillary and tubular structures consistent with ITPN; the cells were uniformly high-grade and lacked mucin production (**Fig. 5A** and **5B**). Immunohistochemically, tumor cells were positive for CK7 and MUC1, partially positive for MUC6, and negative for MUC2, MUC5AC, trypsin, and BCL10. p53 was positive in only a small proportion of cells (approximately 2%), and the Ki-67 labeling index was 10%. SMAD4 IHC was not performed in this case. The diagnosis of ITPN was established based on the characteristic histological architecture and immunohistochemical profile described above. An associated invasive carcinoma measuring 45 × 40 mm was identified beyond the intraductal component. Lymphatic, venous, and perineural invasion were absent. All resection margins were negative (R0). Although the tumor was densely adherent to the SMV–PV axis intraoperatively, no true venous wall invasion was identified histologically (**Fig. 5C**); the interface showed fibrous adhesion. Lymph node metastases were not observed. The histological treatment effect was classified as grade 1a according to the Japanese classification of pancreatic carcinoma by the Japan Pancreas Society, eighth edition,^3)^ and as grade I according to the Evans classification.^4)^ According to the UICC 8th edition,^2)^ the tumor was classified as ypT3N0M0, Stage IIA.

The patient was discharged on POD 16. Adjuvant chemotherapy was not administered. The patient remained disease-free for 29 months after curative surgery and 38 months after the start of chemotherapy.

## DISCUSSION

ITPN is a relatively new intraductal pancreatic tumor concept proposed in 2009.^1)^ It is rare, accounting for approximately 0.9% of exocrine pancreatic tumors and approximately 3% of intraductal tumors.^1)^ According to the World Health Organization classification, pancreatic ITPN was added to the new intraductal neoplasm subgroup in 2010.^5)^ Its common symptoms include abdominal pain, nausea, anorexia, weight loss, and jaundice; however, up to 31.7% of cases may be asymptomatic. The pancreatic head is the most frequent location (51.7%), followed by the body/tail (28.6%) and diffuse involvement (19.5%).^6)^ The reported 5-year overall survival rate is approximately 77%, without significant difference between invasive and noninvasive ITPN; the prognosis is significantly better than that of conventional pancreatic ductal adenocarcinoma.^7)^

The characteristic imaging findings of ITPN include the “2-tone duct sign”—a low-attenuation intraductal mass with associated ductal dilatation on contrast-enhanced CT or MRI—and the “cork-of-wine-bottle sign” on endoscopic retrograde cholangiopancreatography or MRCP.^8)^ On MRI, diffusion-weighted hyperintensity is typical.^9)^ In our case, the main pancreatic duct was dilated with an abrupt interruption on MRCP. CT/MRI findings were suggestive of the “2-tone duct sign” within the dilated duct. DWI demonstrated hyperintensity, as reported in ITPN.

Although reports are limited, FDG-PET frequently shows an increased uptake in ITPN.^10–12)^ The SUVmax in our patient was 6.1 before therapy and 6.58 after therapy.

Pathologically, ITPN lacks gross mucin production and exhibits intraductal proliferation of cuboidal epithelium with tubular and papillary patterns, often accompanied by necrosis or hemorrhage; cytologic atypia is uniformly high-grade.^1)^ The IHC profile typically shows MUC5AC negativity (in contrast to intraductal papillary mucinous neoplasm), MUC1 positivity (approximately 88%), MUC2 negativity, and MUC6 positivity in 60%–70%.^6,13)^ Radiologically, distinguishing ITPN from ACC can be difficult; ACC may extend along the pancreatic ducts.^14)^ Immunonegativity for trypsin and BCL10 was helpful for excluding ACC,^15)^ which supported the final diagnosis in our case.

Published experience with chemotherapy for pancreatic ITPN remains limited. We summarized previously reported pancreatic ITPN cases in which chemotherapy was administered (**Table 2**).^16–25)^ Most reported cases received chemotherapy as adjuvant therapy after resection, whereas only a small number of cases have described chemotherapy before curative-intent resection. In the present case, gemcitabine plus nab-paclitaxel was discontinued after the first course because of adverse events, and S-1 was subsequently administered for 9 courses. The imaging response to systemic chemotherapy was classified as stable disease according to RECIST version 1.1, and the histopathological treatment effect was minimal. Accordingly, this case should not be interpreted as evidence of a marked antitumor effect of chemotherapy against ITPN. Rather, its clinical significance lies in the fact that disease stability was maintained during systemic chemotherapy, mainly with S-1, without distant metastasis, allowing repeat pathological assessment and subsequent curative-intent resection with SMV–PV axis reconstruction using a CFV graft.

**Table 2:** Reported pancreatic ITPN cases treated with chemotherapy and the present case

Author, year	Age/sex	Timing of chemotherapy	Regimen	Surgical/pathological features	Outcome
Shrestha et al.^16)^, 2025	60/M	Preoperative; post-recurrence	FOLFIRINOX preoperatively; gemcitabine plus nab-paclitaxel after liver metastasis	Pancreatic head ITPN-associated carcinoma; ypT1cN0	No or minimal pathological response; liver metastasis 5.5 months after surgery; alive on chemotherapy 2 years 10 months after diagnosis
Nabrinsky et al.^17)^, 2020	52/F	Neoadjuvant	Gemcitabine plus nab-paclitaxel, 4 cycles	Pancreatic head/uncinate process tumor; ypT3N0M0	Tumor decreased on imaging; resection was performed
Yamamoto et al.^18)^, 2023	59/M	Adjuvant	S-1, 6 months	Pancreatic body ITPN; pT3N1aM0	Recurrence 1 year and 10 months after surgery
Senaha et al.^19)^, 2023	70/F	Adjuvant	S-1, 1 year	Pancreatic head ITPN; pT2N0M0	Disease-free 17 months after surgery
Hyakutake et al.^20)^, 2023	60/M	Adjuvant	S-1, 6 months	Pancreatic tail ITPN; pT3N1bM0	Disease-free 9 months after surgery
Raveh et al.^21)^, 2021	24/M	Adjuvant after resection of recurrent disease	FOLFOX, 10 cycles	Recurrent pancreatic head ITPN with associated invasive carcinoma; negative margins	Disease-free 20 months after surgery
Kosmidis et al.^22)^, 2020	82/M	Adjuvant	Gemcitabine, 6 months	Multifocal ITPN with invasive carcinoma; pT3N0	Hepatic metastasis 18 months after surgery; alive with disease 28 months after surgery
Ko et al.^23)^, 2019	61/M	Adjuvant	S-1, 6 months	Pancreatic head ITPN; pT1N0M0	Local recurrence 16 months after surgery; no recurrence 23 months after second surgery
Kölby et al.^24)^, 2015	42/M	Adjuvant	Gemcitabine plus capecitabine, 6 months	Multifocal ITPN; pT3N0M0	Disease-free 19 months after surgery
Urata et al.^25)^, 2012	78/F	Adjuvant	S-1, 6 months	ITPN-associated microinvasive adenocarcinoma with BRAF p.V600E mutation	Postoperative recurrence 34 months after tumor resection
Present case	64/M	Preoperative systemic chemotherapy before curative-intent resection	Gemcitabine plus nab-paclitaxel, 1 course; followed by S-1, 9 courses	Invasive pancreatic head ITPN; ypT3N0M0; SMV–PV axis resection and CFV graft reconstruction	Stable disease according to RECIST version 1.1; disease-free 29 months after surgery and 38 months after chemotherapy initiation

Reported pancreatic ITPN cases treated with chemotherapy are summarized. Published cases are arranged according to chemotherapy timing; the present case is shown separately in the final row.

CFV, common femoral vein; F, female; FOLFOX, folinic acid, fluorouracil, and oxaliplatin; ITPN, intraductal tubulopapillary neoplasm; M, male; RECIST, Response Evaluation Criteria in Solid Tumors; SMV, superior mesenteric vein; PV, portal vein

The timing of surgery was determined by multidisciplinary reassessment after systemic chemotherapy. Although a modest decrease in tumor size was observed, the imaging response to systemic chemotherapy was classified as stable disease according to RECIST version 1.1, and tumor markers were not useful for biological response assessment. However, the absence of distant progression, preserved general condition, repeat EUS-FNA findings suggestive of ITPN, and reassessed technical feasibility of curative-intent resection with planned SMV–PV axis reconstruction supported the decision to proceed with surgery. Continued chemotherapy was also considered; however, the benefit of prolonged chemotherapy for ITPN remains unestablished. Moreover, further treatment could have resulted in cumulative toxicity or loss of the opportunity for curative-intent resection due to future disease progression. Therefore, curative-intent resection was selected after multidisciplinary discussion.

Autologous femoral vein grafting has been reported to be useful for SMV–PV reconstruction during pancreatectomy, particularly when long-segment SMV–PV axis resection is required, because the femoral vein provides an appropriate graft diameter for mesentericoportal venous reconstruction.^26)^ In the present case, the great saphenous vein was inadequate in diameter; therefore, a 3-cm segment of the right CFV was harvested and used for SMV–PV axis reconstruction. Although the resected venous segment measured approximately 5 cm, a 3-cm graft was sufficient because the remaining gap could be approximated after venous mobilization. The CFV was selected because its diameter was considered suitable for the resected SMV–PV axis segment, while the SFJ was preserved to reduce the risk of donor-limb venous congestion. The proximal and distal donor venous stumps were each closed with double-layer over-and-over sutures using 5-0 polypropylene (Prolene). In the present case, IPC with a foot pump was started immediately after surgery, heparin was administered from POD 1, and anticoagulation was switched to edoxaban from POD 5. Although postoperative contrast-enhanced CT detected thrombosis of the right deep femoral vein, no clinically significant lower-limb edema occurred. Edoxaban was continued for 1 year, and follow-up CT 1 year after surgery confirmed resolution of the thrombus.

This case highlights the importance of repeat pathological assessment and multidisciplinary re-evaluation in pancreatic tumors initially managed as pancreatic ductal adenocarcinoma. Although the imaging response to systemic chemotherapy and the histopathological treatment effect were limited, disease stability without distant metastasis provided an opportunity for repeat reassessment and supported the decision to proceed with curative-intent resection with SMV–PV axis reconstruction. Therefore, in selected patients with suspected ITPN, curative-intent surgery should be reconsidered when disease control is maintained and technically feasible resection can be planned.

## CONCLUSIONS

Herein, we report a case of pancreatic ITPN in which disease stability during systemic chemotherapy provided an opportunity for repeat pathological assessment and subsequent curative-intent resection with SMV–PV axis resection and reconstruction. Although the role of perioperative chemotherapy in ITPN remains unclear, this case highlights the importance of multidisciplinary re-evaluation during stable disease, particularly when an unusual histology such as ITPN is suspected and technically feasible resection can be planned.

## DECLARATIONS

### Availability of data and materials

The authors declare that the data used in this study are available within the article.
